# Borderline ovarian clear cell tumor arising from endometriosis during long-term dienogest therapy: a case report

**DOI:** 10.3389/fonc.2025.1732665

**Published:** 2025-12-17

**Authors:** Kaiyue Shang, Hongxin Xing, Suzhen Zhang, Yazhou Zhang, Hui Zhang

**Affiliations:** 1 Department of Obstetrics and Gynaecology, Shandong Provincial Hospital Affiliated to Shandong First Medical University, Jinan, China; 2 Shandong Key Laboratory of Reproductive Research and Birth Defect Prevention, Shandong First Medical University, Jinan, Shandong, China; 3 Department of Ultrasound, Shandong Provincial Hospital Affiliated to Shandong First Medical University, Jinan, China; 4 Department of Pathology, Shandong Provincial Hospital Affiliated to Shandong First Medical University, Jinan, China

**Keywords:** dienogest, endometriosis, ovarian borderline clear cell tumor, ovarian chocolate cysts, ovarian endometrioma

## Abstract

Endometriomas, commonly known as ovarian chocolate cysts, are a prevalent condition in women of reproductive age. They are cysts formed by the ectopic growth of endometrial tissue within the ovary, often leading to dysmenorrhea and infertility. Dienogest is a first-line medication for the long-term management of endometriomas, control of associated pain, and prevention of postoperative recurrence. A 34-year-old woman presented to our hospital with dysmenorrhea and was diagnosed with an endometrioma. She subsequently commenced dienogest treatment for a total duration of thirty-two months. During this period, the cyst gradually decreased in size and eventually became undetectable. However, a follow-up ultrasound indicated the recurrence of the endometrioma after twenty-seven months. Five months later, a subsequent ultrasound revealed papillary growth with internal blood flow within the ovarian endometrioma. The patient underwent immediate surgical intervention. The postoperative pathology indicated a borderline clear cell tumor. Consequently, the patient promptly underwent comprehensive staging surgery, with the final pathology confirming no residual tumor. This case demonstrates that despite long-term and effective dienogest treatment, endometriomas retain the potential for malignant transformation. Therefore, regular monitoring during treatment and prompt intervention upon suspicion of malignancy are indispensable.

## 1 Introduction

Ovarian endometriosis is a common condition among women of reproductive age and a well-recognized precursor of ovarian cancer, particularly associated with clear cell carcinoma and endometrioid carcinoma (1, 2). Dienogest, a highly effective progestin, is widely used for managing endometriosis-related pain and suppressing disease progression (3). However, cases of malignant transformation of endometriomas during effective medical treatment are rarely reported. From the Pubmed search, only a few cases of clear cell carcinoma arising from endometriomas during dienogest therapy have been documented, and no reports have described the development of a borderline clear cell tumor under such conditions (4–6). This case aims to raise awareness among clinicians about this rare yet serious outcome and to emphasize the importance of timely intervention upon detecting signs of malignant transformation to prevent disease progression.

## 2 Case presentation

**Figure 1:**
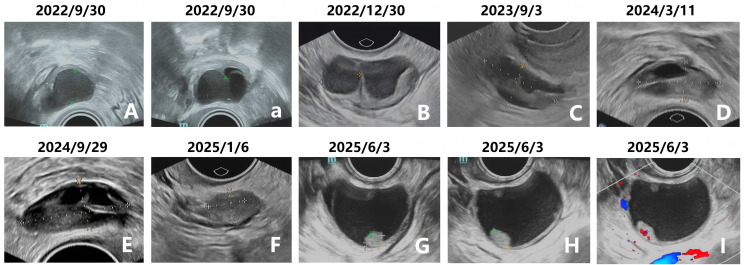
Serial transvaginal ultrasound monitoring of ovarian endometriomas during dienogest therapy. **(A)** Baseline ultrasound (September 2022) shows an endometrioma in the right ovary. **(a)** Baseline ultrasound (September 2022) shows another endometrioma in the right ovary. **(B)** Significant regression of cysts after 3 months of treatment (December, 2022). **(C)** Complete sonographic resolution after 12 months of continuous therapy (September, 2023). **(D)** No evidence of recurrence on follow-up ultrasound (March, 2024). **(E)** Continued absence of recurrence (September, 2024). **(F)** Recurrence of a right ovarian endometrioma at 27 months of therapy (January, 2025). **(G–I)** Rapid progression to a complex cyst with papillary projections and internal vascularity within 5 months (June, 2025).

We present the case of an otherwise healthy 37-year-old woman of East Asian descent with a decade-long history of dysmenorrhea and no family history of malignancies. She had delivered two children vaginally before the age of 30 and had completed her childbearing. At age 34, She commenced treatment with dienogest (2 mg/day) as the first-line and sole therapeutic agent for symptomatic bilateral ovarian endometriomas diagnosed via transvaginal ultrasound (**Figure 1A**). The cysts measured 3.3 × 2.8 × 2.3 cm in the left ovary, while the right ovary contained two cysts measuring 2.4 × 2.4 × 1.7 cm and 2.1 × 2.0 × 1.8 cm (**Table 1**). The patient exhibited an excellent initial therapeutic response. The endometriomas showed significant regression within three months (**Figure 1B**) and became sonographically undetectable after one year of continuous treatment (**Figure 1C**) and subsequent ultrasound examinations over the following year demonstrated no evidence of recurrence (**Figures 1D, E**).

**Table 1:** Serial transvaginal ultrasound monitoring of bilateral ovarian endometriomas during 32-month dienogest therapy.

Date	Left ovarian cyst dimensions (cm)	Volume (cm³)	Right ovarian cyst dimensions (cm)	Volume (cm³)
2022/9/30	3.3 × 2.8 × 2.3	11.1	Cyst 1: 2.4 × 2.4 × 1.7 Cyst 2: 2.1 × 2.0 × 1.8	5.1/4.0
2022/12/30	Not detected	–	Cyst 1: 2.0 × 1.8 × 1.4 Cyst 2: 1.6 × 1.3 × 1.3	2.6/1.4
2023/9/3	Not detected	–	Not detected	–
2024/3/11	Not detected	–	Not detected	–
2024/9/29	Not detected	–	Not detected	–
2025/1/6	Not detected	–	1.2 × 1.1 × 0.9	0.6
2025/6/3	Not detected	–	4.0 × 4.1 × 3.1	26.6

Cyst dimensions are presented in length×width×height (cm). Volumes were calculated using the prolate ellipsoid formula (π/6×L×W×H).

**Figure 2:**
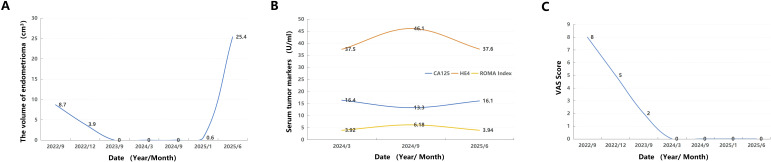
**(A)** Dynamic changes in serum tumor marker levels during clinical monitoring. **(B)** Dynamic changes in serum tumor marker levels during clinical monitoring. **(C)** Longitudinal Follow-up of Dysmenorrhea VAS Scores During Dienogest Therapy.

Notably, after 27 months of uninterrupted dienogest therapy, a right ovarian endometrioma recurred (**Figure 1F**). This lesion demonstrated rapid progression, enlarging to 4.0 × 4.1 × 3.1 cm over the subsequent five months (**Figure 1G**). Critically, follow-up ultrasound revealed suspicious features, including papillary projections with internal vascularity on color Doppler imaging (**Figures 1H, I**). The volume changes of endometrioma is presented in **Figure 2A**. Serum levels of tumor markers, including CA125, HE4, CA19-9, CEA and AFP, remained within normal limits throughout this period (**Figure 2B**). Preoperative laboratory investigations, including complete blood count, urinalysis, coagulation profile, liver function tests, electrocardiogram, abdominal ultrasound, and pelvic CT, all showed normal findings in this patient. The patient’s dysmenorrhea vas scores demonstrated a sustained downward trend and remained at stable low levels (**Figure 2C**).

**Figure 3:**
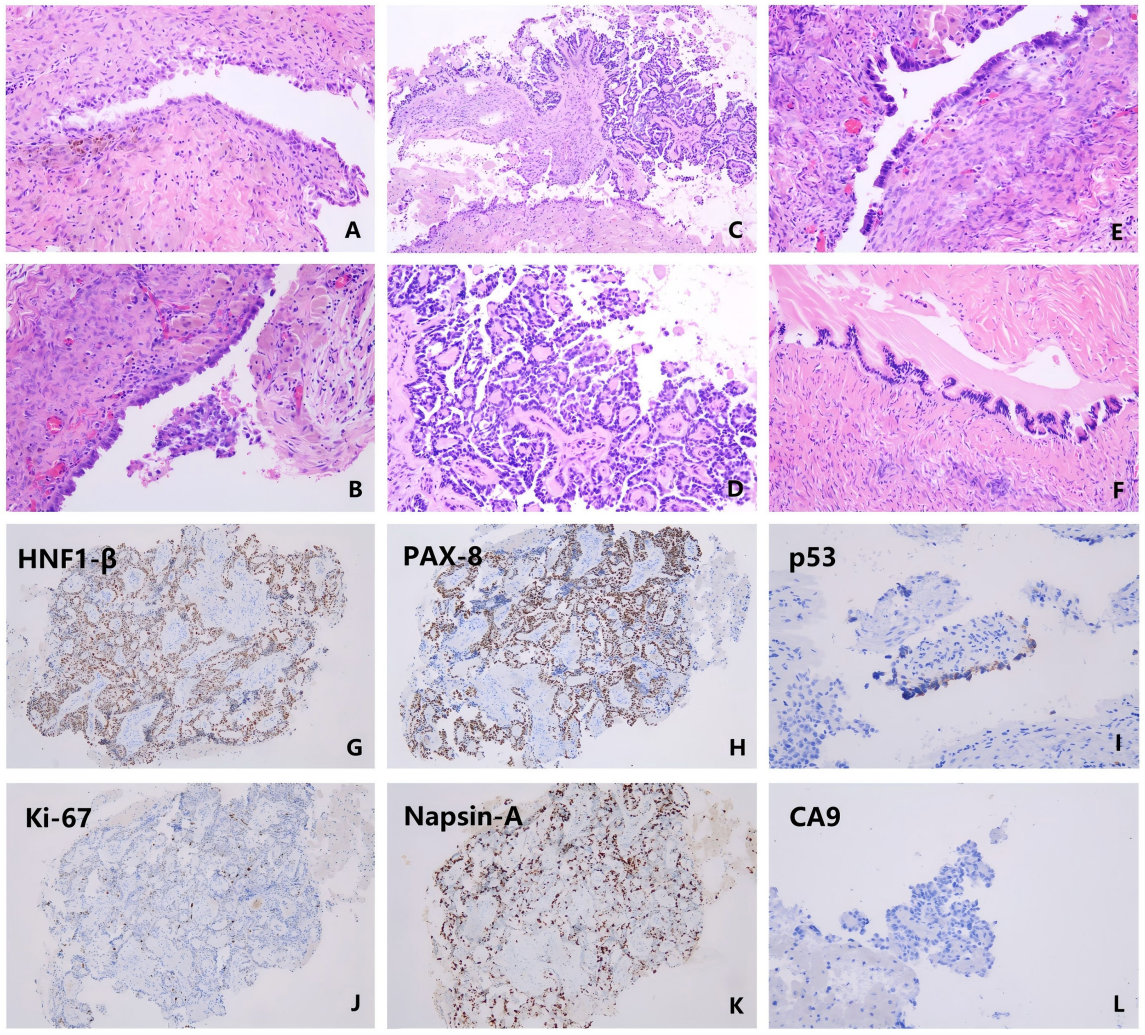
Histopathological characteristics of the resected ovarian lesion (hematoxylin and eosin staining) and diagnostic immunohistochemical profile of the borderline clear cell tumor. **(A)** Endometriotic cyst wall with hemosiderin-laden macrophages, indicating old hemorrhage (×100). **(B)** Borderline clear cell tumor with intracystic growth pattern (×200). **(C)** Borderline clear cell tumor demonstrating papillary projections (×100). **(D)** Borderline clear cell tumor with characteristic clear cytoplasm and nuclear atypia (×200). **(E)** Serous cystadenoma (×200). **(F)** Mucinous cystadenoma showing tall, mucin-containing epithelial cells (×200). **(G)** Strong nuclear positivity for HNF1-β. **(H)** Diffuse nuclear staining for PAX-8. **(I)** Wild-type p53 staining pattern. **(J)** Ki-67 proliferation index of approximately 40%. **(K)** Negative for Napsin-A. **(L)** Negative for CA9.

Laparoscopic right ovarian cystectomy was performed. Postoperative pathology confirmed a right ovarian endometriotic cyst (**Figure 3A**) with focal papillary architecture (**Figures 3B, C**), consistent with a borderline clear cell tumor (**Figure 3D**), and accompanied by focal seromucinous cystadenoma (**Figures 3E, F**). The diagnosis was confirmed by a characteristic immunohistochemical profile. The tumor cells were positive for HNF1-β (**Figure 3G**) and PAX-8 (**Figure 3H**), but negative for WT-1 and ER. They exhibited a wild-type p53 pattern (**Figure 3I**) and a Ki-67 proliferation index of approximately 40% (**Figure 3J**). Furthermore, the tumor was negative for Napsin-A (**Figure 3K**) and CA9 (**Figure 3L**), with only focal positivity for P16. An intact nuclear expression of all mismatch repair proteins (MLH1, PMS2, MSH2, MSH6) was also observed. Given the patient’s completed family planning, she subsequently underwent definitive surgical staging via total hysterectomy, bilateral salpingo-oophorectomy, omentectomy and multiple peritoneal biopsies. The final pathology confirmed a FIGO stage IA tumor with no residual disease. The patient remains disease-free during four months of postoperative surveillance, with follow-up gynecological ultrasound, CA125, CA199, HE4, and ROMA index all within normal limits; she has not received hormone replacement therapy to date. This case highlights that while dienogest is effective in managing endometrioma-related symptoms, it does not entirely eliminate the risk of malignant transformation.

## 3 Discussion

The management of ovarian endometriomas is a systematic process that requires an individualized approach based on factors such as the patient’s age, symptoms, fertility desires, cyst size, and previous treatment history (7). First-line hormonal therapies—including combined oral contraceptives, progestins, and GnRH agonists with add-back therapy—effectively suppress pain and disease progression for most patients. For those with refractory symptoms or large cysts, laparoscopic cystectomy remains the definitive surgical intervention (8). When patients have small cysts (typically <5 cm in diameter), no immediate fertility plans, or prefer to avoid surgery, pharmacological treatment can be chosen (9). The goal of such treatment is to suppress ovarian function, halt menstruation, and induce a “pseudo-pregnancy” or “pseudo-menopausal” state, thereby promoting the atrophy of ectopic endometrial tissue and alleviating pain (10). Dienogest is a first-line recommended medication for ovarian endometriomas (11), proven to effectively reduce cyst size, with the advantages of high efficacy, good tolerability, and suitability for long-term use (3). However, rare adverse reactions to dienogest have also been reported in the literature, including dysmenorrhea, dyspepsia, lower abdominal pain, urticaria, and peritonitis (12).This case clearly demonstrates both the effectiveness of dienogest in suppressing the growth of ectopic endometrium and its limitation in being unable to completely prevent malignant transformation.

The role of hysterectomy in the management of borderline ovarian tumors remains unclear. In postmenopausal women with borderline ovarian tumors, hysterectomy is associated with a reduced risk of recurrence without affecting all-cause mortality or disease-specific mortality (13). However, current evidence does not support routine hysterectomy for all patients with borderline ovarian tumors. Although uterine-sparing surgery may increase the risk of recurrence, it does not elevate disease-related mortality or all-cause mortality (14). For young women with early-stage borderline ovarian tumors, utero-ovarian preservation may be associated with improved overall survival compared to ovarian preservation alone, without compromising borderline ovarian tumor-related survival outcomes (15). In our case, the patient’s decision to undergo hysterectomy was based on her completed family planning goals and the desire for definitive surgical management following the diagnosis of a borderline clear cell tumor. This approach aligned with her preference to minimize future cancer risks and avoid potential diagnostic challenges associated with monitoring both ovarian and uterine health post-treatment.

Numerous studies have indicated that women with endometriosis have an increased risk of developing ovarian malignancies (16). A retrospective case-control study revealed that advanced age, menopause, weight loss, cyst diameter ≥8.33 cm, and the presence of solid components on ultrasound are noteworthy risk factors for endometriosis-associated ovarian cancer (17). However, during dienogest therapy, cysts are typically well-controlled and remain small in volume (18). Oral contraceptive progestins appear to exert a protective effect against ovarian cancer (19). As a progestin, dienogest is theoretically capable of preventing the malignant transformation of endometriosis. We identified only five cases in PubMed reporting malignant transformation of endometriosis during long-term dienogest use (4, 5), with all cases pathologically confirmed as clear cell carcinoma. Notably, the cysts in these reported cases of malignant transformation during dienogest treatment were not large. In our case, the patient was young, premenopausal, and had a small cyst diameter, yet still experienced malignant transformation.

The immunohistochemical findings in this case hold classic diagnostic significance and were pivotal in confirming the diagnosis of a borderline clear cell tumor. The tumor cell expression of HNF1-β and PAX-8 serves as a highly specific immunomarker combination for ovarian clear cell neoplasms, providing cornerstone evidence for the diagnosis (20). The negative expression of WT-1 and ER effectively excludes more common ovarian carcinoma types, such as high-grade serous carcinoma (typically WT-1+/ER+) and endometrioid carcinoma (typically ER+) (21, 22). The P53 wild-type expression pattern rules out a TP53-mutant high-grade carcinoma, which is consistent with the tumor’s low malignant potential (23). In the papillary areas, the Ki-67 proliferation index of approximately 40% precisely reflects the “borderline” nature of the lesion. It is higher than that typically seen in benign tumors yet lower than the Ki-67 index (often >80%) observed in invasive clear cell carcinomas, indicating a tumor with progression potential that has not yet reached a fully malignant state (24). The intact expression of mismatch repair proteins (MLH1, PMS2, MSH2, MSH6) indicates a microsatellite-stable tumor, aligning with the profile of most sporadic ovarian tumors (25).

Notably, in this case, serum tumor markers such as CA125 remained within normal limits despite malignant transformation, but studies have shown that combining systemic inflammatory indicators, such as the Systemic Inflammation Response Index (SIRI) and Systemic Inflammation Response (SIR), with CA-125 reveals significantly elevated SIR-125 and SIRI-125 values in ovarian cancer patients compared to those with borderline ovarian tumors. This combined approach enhances diagnostic discrimination between ovarian cancer and borderline ovarian tumors, offering a simple and cost-effective preoperative tool (26). This case underscores the critical role of regular pelvic ultrasound in monitoring patients. Features such as cyst recurrence, rapid enlargement over a short period, or the development of solid components, papillary structures, and internal blood flow should raise strong suspicion for malignancy (27). Beyond conventional ultrasound, advanced diagnostic methodologies are enhancing our capacity for the early detection of malignant transformation in endometriosis. The research conducted by Camelia Alexandra Coadă et al. identified 14 miRNAs that demonstrate progressively increasing expression levels from ovarian endometriosis to transitional lesions, ultimately leading to endometriosis-associated ovarian cancer. This finding provides valuable insights into the molecular progression from benign to malignant lesions and suggests potential biomarkers for the early detection of endometriosis-associated ovarian cancer (28). Moreover, MR relaxometry has emerged as a promising adjunctive tool. Using a 3 Tesla MR system with a multi-echo sequence, the transverse relaxation rate (R2) can be quantified, with a value below 12.1 s^−1^ suggesting malignancy (29). This technique is particularly valuable for fertility-sparing counseling, as endometriotic-cyst-associated ovarian cancer typically presents as a unilocular cyst with T2-hypointense content, a feature distinguishable by MRI (30). The role of iron metabolism in malignant transformation is further underscored by the significantly lower total iron levels in endometriotic-cyst-associated ovarian cancer cysts (14.2 ± 36.6 mg/L) compared to benign endometriotic cysts (244.4 ± 204.9 mg/L; p < 0.001), establishing iron-related compounds as biomarkers of high diagnostic value (31). Looking forward, machine learning algorithms are being leveraged to develop sophisticated risk prediction models for forecasting malignant transformation, representing the next frontier in personalized patient management (32).

## 4 Conclusions

Dienogest is an effective medication for managing endometriosis. However, it does not exempt patients from the risk of malignant transformation. This case strongly recommends continuous and rigorous imaging surveillance for patients with endometriomas undergoing long-term medical therapy, as it is crucial for the early detection of malignant transformation, enabling timely intervention and improving clinical outcomes.

## Data Availability

The original contributions presented in the study are included in the article/supplementary material, further inquiries can be directed to the corresponding author/s.
